# Hypoglycemic conditions enhance neonatal β-cell glucose responsiveness via AMPK/mTOR-increased K_ATP_ channel density

**DOI:** 10.1210/endocr/bqag085

**Published:** 2026-07-31

**Authors:** Ekaterina Novichkova, Haiqiang Dou, Nimisha Gupta, Polina Kotova, Johan Tolö, Caroline Miranda, Patrik Rorsman, Aharon Helman, Michael D Walker

**Affiliations:** Department of Biomolecular Sciences, Weizmann Institute of Science, Rehovot 7610001, Israel; Institute of Neuroscience and Physiology, Department of Physiology, Metabolic Research Unit, Sahlgrenska Academy, Box 432, University of Gothenburg, 405 30 Gothenburg, Sweden; Department of Biomolecular Sciences, Weizmann Institute of Science, Rehovot 7610001, Israel; Department of Biochemistry, Food Science and Nutrition, The Robert H Smith Faculty of Agriculture, Food and Environment, The Hebrew University of Jerusalem, Rehovot 7610001, Israel; Institute of Neuroscience and Physiology, Department of Physiology, Metabolic Research Unit, Sahlgrenska Academy, Box 432, University of Gothenburg, 405 30 Gothenburg, Sweden; Institute of Neuroscience and Physiology, Department of Physiology, Metabolic Research Unit, Sahlgrenska Academy, Box 432, University of Gothenburg, 405 30 Gothenburg, Sweden; Institute of Neuroscience and Physiology, Department of Physiology, Metabolic Research Unit, Sahlgrenska Academy, Box 432, University of Gothenburg, 405 30 Gothenburg, Sweden; Oxford Centre for Diabetes, Endocrinology and Metabolism, Radcliffe Department of Medicine, University of Oxford, Oxford OX3 7LJ, UK; Department of Biochemistry, Food Science and Nutrition, The Robert H Smith Faculty of Agriculture, Food and Environment, The Hebrew University of Jerusalem, Rehovot 7610001, Israel; Department of Biomolecular Sciences, Weizmann Institute of Science, Rehovot 7610001, Israel

**Keywords:** glucose responsiveness, K_ATP_ channel, insulin secretion, neonatal hypoglycemia, trafficking, mTOR

## Abstract

At birth, blood glucose levels drop sharply before rising to normoglycemic adult levels. This is accompanied by functional maturation of pancreatic β-cells and the acquisition of glucose-responsive insulin secretion. Although transcriptional programs have been well characterized, the metabolic adjustments that enhance insulin secretion in neonatal β-cells remain poorly understood. Neonatal islets exhibit a high rate of basal insulin secretion, and elevating glucose to high concentrations produces little additional stimulation (unlike adult islets). This study aimed to elucidate how neonatal pancreatic β-cells become glucose-responsive, using an in vitro protocol involving culture of neonatal islets at low glucose to emulate systemic hypoglycemia. Low-glucose exposure markedly improved glucose responsiveness of neonatal islets by selectively lowering insulin secretion at low glucose concentrations. Unlike adult β-cells, neonatal β-cells were depolarized and generated spontaneous action potentials at low glucose, resulting in elevated [Ca^2+^]_i_ and a blunted response to high glucose. These changes were reversed by brief (1-2 hours) low-glucose culture, which was accompanied by increased K_ATP_ channel surface expression and activity. The effects of low-glucose incubation were mimicked by the AMPK agonist AICAR. These insights may inform strategies to restore glucose-regulated insulin secretion in type 2 diabetes.

In adult pancreatic β-cells, nutrient influx is tightly coupled to insulin secretion (1). In addition to glucose, the major physiological regulator of insulin release (2), nutrients such as long-chain fatty acids and amino acids, and hormones such as GLP-1 and glucocorticoids fine-tune insulin secretion (3). Glucose-induced insulin secretion (GIIS) in pancreatic β-cells is regulated by a network of metabolic sensors and ion channels. At the core of this system is the ATP-sensitive potassium (K_ATP_) channel, which couples metabolic state to membrane excitability and insulin release (4). Glucose sensing in the β-cell depends critically on the intracellular balance between ATP and ADP (5). Glucose entry into β-cells elevates the intracellular ATP/ADP ratio, thereby lowering K_ATP_ channel activity (6). This results in membrane depolarization, which in turn activates plasmalemmal voltage-dependent calcium channels, allowing calcium influx (7), thereby triggering the fusion of insulin granules with the plasma membrane and the release of insulin (8).

Embryonic and neonatal β-cells exhibit a distinct functional state marked by limited responsiveness to glucose (9). During early postnatal development, these cells gradually acquire the molecular and metabolic features required for glucose-stimulated insulin secretion (GIIS) (10-12). Notably, K_ATP_ channel activity is low in embryonic β-cells (13), lowering the threshold for membrane depolarization and insulin secretion (14). The signals and mechanisms that control β-cell maturation and the development of GIIS, as well as the precise molecular events underlying this transition, remain poorly understood (15).

At birth, blood glucose concentrations decline substantially before gradually rising toward adult levels. Although this transitional period is generally considered a physiological adaptation to extrauterine life, the distinction between physiological transitional hypoglycemia and pathological neonatal hypoglycemia remains a matter of ongoing discussion (16). During this period of rapid growth, insulin demand is high despite limited glucose availability. How β-cells adapt insulin secretion to these competing demands remains unresolved. Factors such as nutrient availability, oxygen tension, and hormonal signals have been proposed to influence postnatal β-cell function (17, 18). However, whether and how changes in glucose availability directly modulate the functional state of β-cells remains unclear. Here, we investigate how metabolic conditions acutely regulate insulin secretion and stimulus-secretion coupling in neonatal β-cells, focusing on mechanisms that define their functional state. These studies have broader pathophysiological implications, as evidence indicates that β-cells revert to a less mature state in diabetes (19). Understanding how the functional state of glucose-responsive insulin secretion is dynamically and reversibly regulated is critical, as loss or impairment of β-cell glucose responsiveness is a hallmark of type 2 diabetes.

## Materials and methods

### Animals

Experiments with adult mice used 8- to 12-week-old C57BL/6 mice. Experiments with neonatal and embryonic mice used 24-hour-old (P1) and E18 ICR(CD1) pups and embryos. Mice were obtained from the Weizmann Institute animal facility (Rehovot, Israel) and from Charles River Laboratories (Germany). All procedures were approved by the local Ethics Committees and conducted in accordance with institutional and national guidelines.

### Islet isolation and functional assays

We isolated islets from adult pancreases by injecting collagenase P (Roche) into the bile duct, followed by isolation on Histopaque 1119 and 1077 gradients (Sigma). For neonatal and embryonic islet isolation, pancreases from P1 pups or E18 embryos were microdissected, grouped, and digested with collagenase. Islets were purified by Histopaque 1077 density-gradient separation, handpicked, and allowed to recover overnight in complete RPMI 1640 medium before incubation for 20-24 hours in RPMI containing 3 mM (low) or 11 mM (high) glucose. Insulin secretion was measured in KRBH buffer for 1 hour with glucose ± AICAR or ± diazoxide. Insulin secretion was quantified using the HTRF Insulin Ultra Sensitive Kit (Cisbio). Each biological replicate consisted of nine islets grouped from 3-4 animals. Each condition was tested in ≥ 3 biological replicates.

### Flow cytometry

Islet cells were dissociated, fixed, permeabilized, and stained for insulin, glucagon, pAMPK, and phospho-S6 as previously described, with minor modifications (20). Antibodies used: Anti-insulin: Agilent Cat# A0564, RRID:AB_10013624; Anti-Phospho-S6 Ribosomal Protein: Cell Signaling Technology Cat# 5364, RRID:AB_10694233; Anti-AMPK alpha 1

(phospho T183) + AMPK alpha 2 (phospho T172): Abcam Cat# ab194920, RRID:AB_3751526. Data were acquired on a CytoFLEX flow cytometer (Beckman Coulter) and analyzed using FlowJo v10.8 (BD Life Sciences).

### RNA extraction and RNA-seq

Total RNA was extracted from islets using the NucleoSpin RNA XS kit (Macherey-Nagel) according to the manufacturer's protocol. Bulk RNA-seq of P1 and adult islets was performed using the MARS-seq pipeline (21). Library preparation and sequencing were carried out at the G-INCPM core facility (Weizmann Institute), and data were visualized in R using ggplot2.

### Electrophysiology

Membrane potential and currents were recorded from cells in intact islets using an EPC-10 USB amplifier and PatchMaster software (HEKA Electronic, Germany). Recordings were performed using the perforated-patch or ruptured whole-cell configurations as previously described (22). Amphotericin B (240 µg/mL) was used for perforation. Cells were superfused with standard extracellular solution. For determination of K_ATP_ channel density, the standard whole-cell configuration was used, and the pipette solution contained ATP/ADP (Mg^2+^ salts; 0.1 and 0.3 mM, respectively). Cell identity was verified by electrophysiological fingerprinting (23). Data were analyzed using Fitmaster v2 × 90.2 (HEKA Electronic) and Igor 6 (WaveMetrics, OR, USA).

### Calcium imaging

Changes in intracellular Ca^2+^ were recorded in individual β-cells of intact islets loaded with the calcium indicator Fluo-4 AM (4 μM; Invitrogen, F14201). Islets were loaded with the ester for 90 minutes at 37 °C, then imaged (after formation of free Fluo-4) in an RC-25 chamber (Warner Instruments) on a Ti2 inverted microscope (Nikon) (see Electrophysiology). The fluorophore was excited at 488 nm, and the emitted light was collected with an EMCCD camera (iXon 897 Ultra, Andor) at 34 °C. Data were processed in (https://fiji.sc) (24) after background subtraction to calculate ΔF/FG3.

### Statistical analysis

Statistical analyses were performed using GraphPad Prism (GraphPad Software, San Diego, CA, USA). Data are presented as means ± SEM from ≥3 biological replicates. Two-tailed unpaired *t*-tests were used, with significance set at *P* ≤ .05 (*), *P* ≤ .01 (**), *P* ≤ .001 (***), and *P* ≤ .0001 (****). For [Ca^2+^]i measurements, data were analyzed with linear mixed-effects models (lme4 R package) (25) with islet as a random effect. Post hoc pairwise comparisons used Tukey HSD correction (emmeans package) (26). When normalized, reference groups had zero variance; therefore, unpaired *t*-tests on raw values were used instead. The Mann–Whitney test was used for nonparametric comparisons.

## Results

### In vitro exposure of neonatal islets to hypoglycemic conditions reduces basal insulin secretion

**Figure 1:**
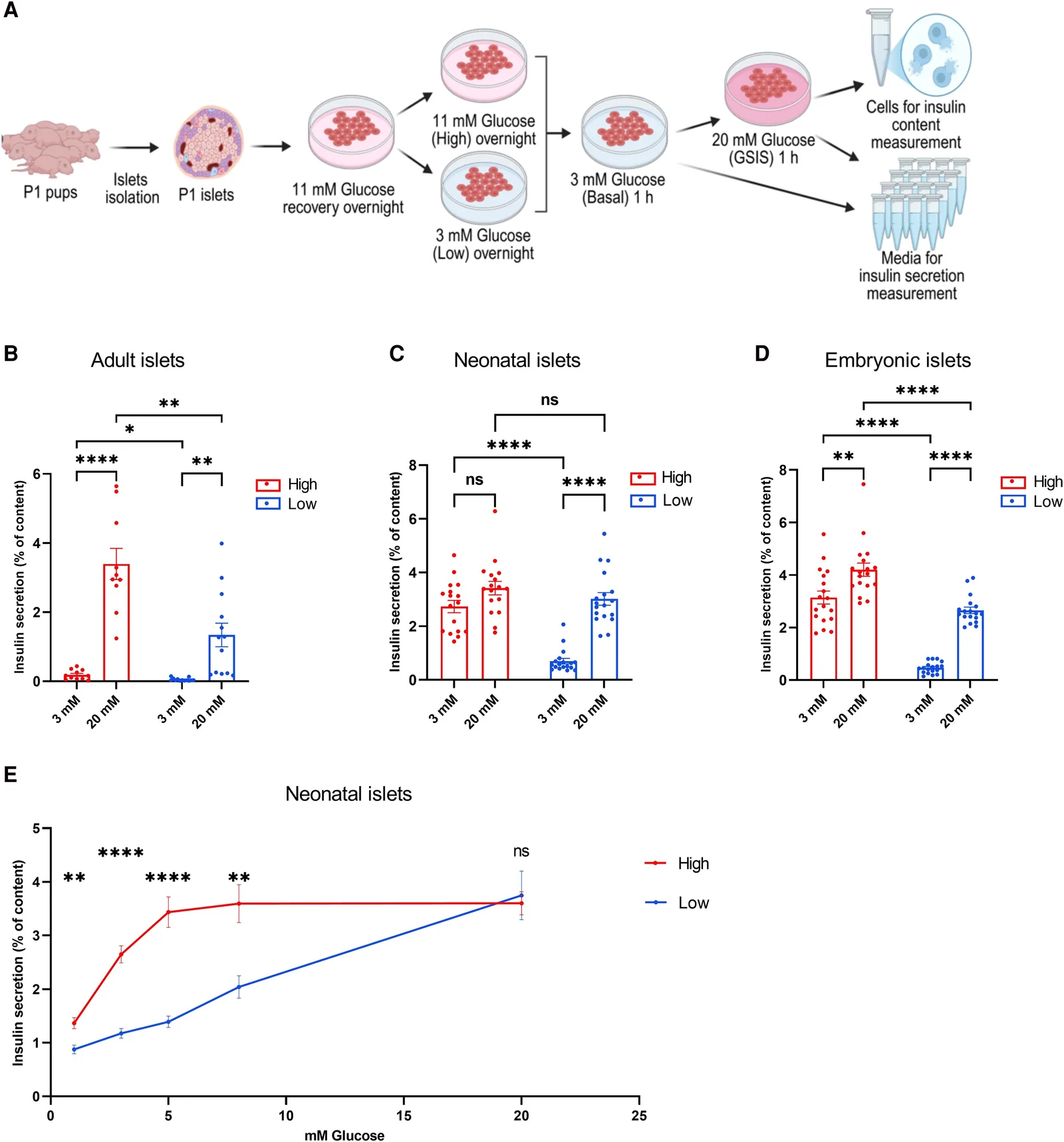
Experimental design and insulin secretion profiles under high glucose and low glucose conditions. (A) Schematic representation of the experimental setup used to study the effect of low glucose on insulin secretion in mouse islets. Islets from 3-month-old adult, 1-day-old mice, and E18 embryos were incubated in 3 mM glucose (pretreated low glucose) or 11 mM glucose (pretreated high glucose) in RPMI medium for 24 hours. The islets were then sequentially challenged with 3 mM (Basal) and 20 mM (GIIS) glucose in KRBH medium for 1 hour and insulin secretion was determined. (B-D) Insulin secretion by (B) adult (*n* = 10), (C) P1 (*n* = 18) and (D) embryonic (*n* = 18) islets, pretreated with high- or low-glucose. Relative insulin secretion is expressed as a percentage of total insulin content. Each data point represents insulin secretion from a group of 9-10 islets. Results were confirmed in at least three independent experiments. (E) Insulin secretion by neonatal (P1) mouse islets after 24 hours preincubation in RPMI medium under high glucose or low glucose conditions, followed by stimulation with increasing concentrations of glucose (1,3,5,8 or 20 mM) in KRBH medium for 1 hour. Insulin secretion is expressed as a percentage of total insulin content. Each insulin measurement was performed in groups of 9 islets (*n* = 12). Results were confirmed in 3 independent experiments. Bars indicate mean ± SEM. Statistical significance was assessed using the Welch ANOVA with the Dunnett multiple comparisons test vs the 24 hours condition. *P < .05, **P < .01, ***P < .001, ****P < .0001. Created in BioRender. Walker, M. (2026) https://BioRender.com/gx2dv40.

We hypothesized that acquisition of β-cell glucose responsiveness may be triggered by neonatal hypoglycemia. Because glucose responsiveness develops gradually during late fetal and early postnatal life, we included both E18 embryonic and P1 neonatal islets. This allowed us to determine whether the effects of low-glucose exposure are a general feature of developmentally immature β-cells rather than a phenomenon restricted to the immediate postnatal period. To test this, we established an in vitro system in which adult and neonatal islets were exposed to conditions mimicking neonatal hypoglycemia (3 mM glucose for 24 hours; “low glucose”) (Fig. 1A) and compared them to an elevated glucose concentration (11 mM glucose; “high glucose”). Exposure of adult islets to 3 mM glucose preserved robust glucose-stimulated insulin secretion, although the magnitude of insulin secretion at 20 mM glucose was reduced after low-glucose pretreatment (Fig. 1B). By contrast, neonatal (Fig. 1C) and embryonic (Fig. 1D) islets exhibited high basal insulin secretion and little or no GIIS. In striking contrast, following exposure to 3 mM glucose, embryonic and neonatal islets showed significantly reduced basal insulin secretion, accompanied by robust GIIS (Fig. 1C, D). We also tested whether pretreatment with low and high glucose affects the response of neonatal islets to amino acids. For this, P1 islets were incubated overnight in low (3 mM) or high (11 mM) glucose medium in the absence of amino acids. Insulin secretion was then measured over the course of 1 hour in KRBH containing 3 or 20 mM glucose in the presence or absence of amino acids added at standard RPMI 1640 concentrations. Whereas high-glucose islets show little or no additional change in insulin secretion in response to amino acids, low-glucose pretreatment led to significant amino acid-dependent insulin secretion, both at high and low glucose concentrations (Fig. S1) (27).

### Exposure of neonatal islets to low glucose conditions rapidly shifts glucose threshold to adult-like state

To test whether the high basal secretion reflects constitutive insulin release or altered glucose sensitivity, we examined insulin secretion across a range of glucose concentrations in neonatal islets exposed to high- and low-glucose conditions. We observed that islets cultured under high-glucose conditions displayed high insulin secretion across the tested glucose range, whereas low-glucose pretreatment reduced insulin secretion at low glucose concentrations and altered the overall glucose-response profile. These findings indicate that prior glucose exposure alters the glucose-response profile of neonatal islets across the tested glucose range, and that exposure to hypoglycemic conditions shifts the range of glucose responsiveness toward higher, adult-like glucose concentrations by increasing the glucose threshold for insulin secretion (28).

### Slow acquisition of glucose competence following low glucose exposure

**Figure 2:**
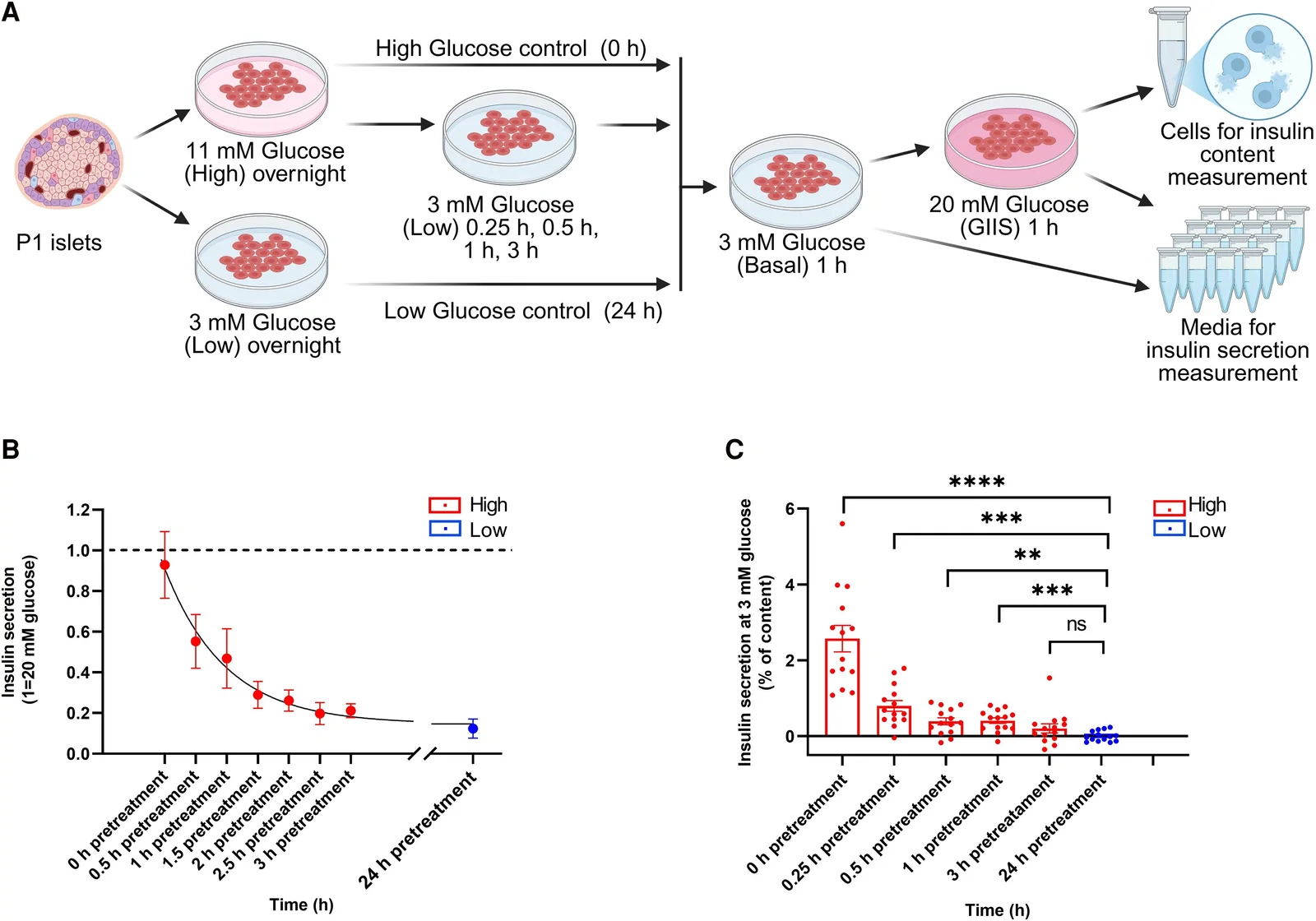
Kinetics of decline in insulin secretion following pre-exposure to low glucose. (A) Schematic representation of the experimental protocol used to assess the kinetics of decline in insulin secretion following pre-exposure to low glucose. P1 mouse islets were pretreated overnight in either high- (11 mM) or low-glucose (3 mM) conditions in RPMI medium. Islets pretreated overnight in high-glucose were subsequently exposed to low-glucose (3 mM) in KRBH for the indicated time intervals (0.25-3 hours). Following this exposure, islets were transferred to fresh low-glucose KRBH, and insulin secretion released during a 1 hour incubation was measured. Islets pretreated overnight in low glucose were transferred directly to fresh low-glucose KRBH for 1 hour and served as the (24 hour) low-glucose control. Islets pretreated overnight in high glucose and transferred directly to low glucose for 1 hour served as the high-glucose (0 hour) control. Insulin released into the medium during these incubations was quantified. (B) Kinetics of decline of basal insulin secretion (3 mM glucose) expressed relative to secretion at 20 mM glucose (=1). Data are plotted against time of exposure to low glucose (indicated as pretreatment intervals) and fitted using a one-phase exponential decay model by nonlinear regression. The dashed horizontal line indicates insulin secretion at 20 mM glucose (=1). Each insulin measurement was performed from groups of 9 islets (*n* = 14). Thus, the point labeled “0 hours pretreatment” has been exposed to 3 mM glucose for 1 hour, the point labeled “0.25 hours pretreatment” has been exposed to 3 mM glucose for 1.25 hours etc. (C) Insulin secretion at 3 mM glucose in neonatal islets following pre-exposure to low glucose for the indicated time intervals. Duration of exposure to 3 mM glucose as defined in panel (B). The 24 hours condition represents islets pretreated in low glucose. Each insulin measurement was performed from a group of 9 islets, *n* = 12. Bars indicate mean ± SEM. Statistical significance was assessed using the Welch ANOVA with the Dunnett multiple comparisons test vs the 24 hours condition. *P < .05, **P < .01, ***P < .001, ****P < .0001. Created in BioRender. Walker, M. (2026) https://BioRender.com/x6p9ah1.

In adult islets, lowering glucose from stimulatory to nonstimulatory concentrations switches off insulin secretion within minutes. We therefore examined whether the suppression of insulin secretion observed in neonatal islets after low-glucose exposure reflects an acute response or a slower adaptive process. Neonatal (P1) mouse islets were cultured overnight in either high-glucose (11 mM) or low-glucose (3 mM) complete RPMI medium. Islets maintained overnight in high-glucose conditions were subsequently exposed to low-glucose (3 mM) KRBH for the indicated time intervals (0.25-3 hours). Basal insulin secretion was then assessed by transferring islets to fresh low-glucose KRBH (3 mM) for 1 hour and measuring insulin released into the medium. GIIS was then assessed during a 1 hour incubation at 20 mM glucose. Islets cultured overnight in low-glucose medium and then transferred directly to low-glucose KRBH for 1 hour served as the low-glucose control, while islets cultured overnight in high-glucose medium and then transferred to low-glucose KRBH for 1 hour served as the high-glucose control (Fig. 2A).

Figure 2B summarizes insulin secretion in high-glucose-incubated islets during 1 hour incubations after prior exposure to low glucose for 0 minutes to 3 hours (in 30-min increments). We plot insulin secretion as a fraction of the value at 20 mM glucose. Notably, basal insulin secretion measured over the first 1 hour after lowering glucose remained as high as in islets continuously exposed to high glucose (Fig. 2B), but then exhibited a gradual, time-dependent decline with a time constant (*τ*) of 1.54 hours. In agreement with these data, we found in a larger data set that insulin secretion at 3 mM glucose in islets incubated overnight at high glucose, in excess of that seen in islets incubated overnight at low glucose, remained elevated for >1 hour, and that it was only after 3 hours that a statistically significant enhancement was no longer detectable (Fig. 2C). Thus, the markedly slower kinetics of insulin secretion reduction observed with neonatal islets exposed to low glucose conditions are clearly distinct from the acute reversal of glucose-induced insulin secretion (29).

### Transcriptomic analysis reveals differential response to low glucose conditions in adult and neonatal islets

**Figure 3:**
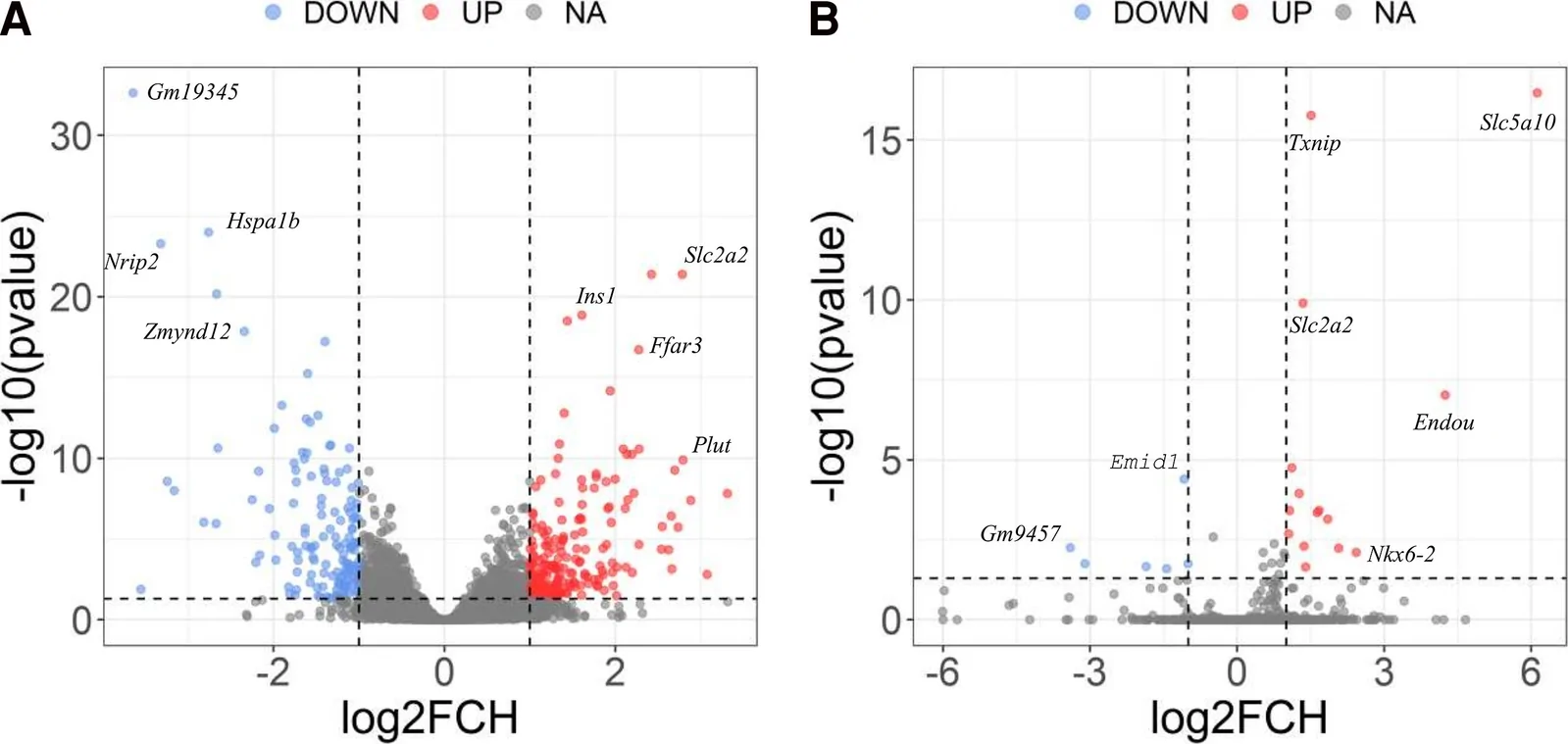
Gene expression changes in adult and neonatal islets after in vitro low- and high-glucose pretreatment. Volcano plots depicting differentially expressed (DE) genes in (A) adult and (B) P1 islets following 24 hours preincubation in RPMI medium containing either 3 mM or 11 mM glucose. Islets from 3-month- and 1-day-old mice were incubated for 24 hours in RPMI medium containing 3 mM or 11 mM glucose. RNA was extracted from ∼100 islets per replicate, and sequenced following the bulk MARS-seq protocol. Approximately 18,000 genes were analyzed. Differential expression analysis was performed using the DESeq2 package in R. Fold change (FCH) was calculated as the ratio of gene expression in the high-glucose condition to that in the low-glucose condition (11 mM/3 mM). *P* values were adjusted for multiple comparisons. Blue points represent genes that were downregulated in high-glucose islets or upregulated in low-glucose islets. Conversely, red points represent genes that were upregulated in the high-glucose state or downregulated in the low-glucose state. The experiment included 5 biological replicates for each condition. One replicate was omitted from the neonatal comparison. Selected representative differentially expressed genes are indicated on the plots.

To elucidate the mechanisms underlying the effects of low glucose on neonatal islets, we performed bulk RNA-seq on adult and neonatal (P1) islets exposed to low- and high-glucose conditions. The corresponding RNA-seq dataset is publicly available in the Gene Expression Omnibus under accession number GSE314732 (30). Whereas in adult islets (Fig. 3A), a substantial number of genes (344) were differentially expressed, a much smaller number of genes (21) were significantly affected in neonatal islets (Fig. 3B).

These results suggest that adult islets undergo robust transcriptional adaptation to changing glucose concentrations in vitro, whereas neonatal islets exhibit an attenuated transcriptional response to low-glucose exposure. Strikingly, key genes involved in islet identity, metabolism, and maturation (*Slc2a2*, *G6pc2*, *Ucn3*, and *Mafa*) were minimally affected in neonatal islets (Table 1). We also observed that expression of the genes encoding K_ATP_ channel subunits, *Kcnj11* (KIR6.2) and *Abcc8* (SUR1), was not significantly altered following low-glucose exposure in either adult or neonatal islets (Table 2). We conclude that the observed functional changes in insulin secretion are unlikely to be transcriptionally mediated.

**Table 1:** Comparison of gene expression changes in adult and neonatal islets following 24 hours exposure to high vs low glucose conditions

	Adult high glucose vs low glucose				P1 high glucose vs low glucose			
Gene	Log2 Fold Change	Abs. Fold Change	*P* adj.	DE	Log2 Fold Change	Abs. Fold Change	*P* adj.	DE
*Slc2a2*	2.3	4.8	0E + 00	yes	1.3	2.5	1E − 10	yes
*G6pc2*	2.8	6.9	0E + 00	yes	1	2	1E − 02	yes
*Ucn3*	1.4	2.6	8E − 07	yes	−1	0.5	6E − 02	no
*Mafa*	2.2	4.4	5E − 08	yes	0.6	1.6	6E − 01	no
*Prlr*	2.9	7.4	5E − 08	yes	1.7	3.2	4E − 04	yes
*Cox6a2*	0.5	1.4	4E − 01	no	1.8	3.6	7E − 04	yes
*Txnip*	0.6	1.5	4E − 01	no	1.5	2.8	2E − 16	yes
*Ffar1*	1.6	2.9	5E − 05	yes	—			
*Ffar3*	2.7	6.3	5E − 07	yes	—			

Fold change was calculated as the ratio of gene expression in the high glucose state to the low glucose state. Threshold for determining differential expression (DE) was set at ±1 for Log2 Fold Change and 0.05 for adjusted *P* value. RNA-seq results for *Slc2a2*, *Prlr* and *Cox6a2* were validated with qPCR (data not shown). Genes with very low absolute expression levels, such as Slc5a10, were excluded from the analysis.

**Table 2:** Expression of K_ATP_ channel subunit genes (*Kcnj11*, *Abcc8*) in islets exposed to high vs low glucose

	Adult high glucose vs low glucose				P1 high glucose vs low glucose			
Gene	Log2 Fold Change	Abs. Fold Change	*P* adj.	DE	Log2 Fold Change	Abs. Fold Change	*P* adj.	DE
*Kcnj11*	−0.14	0.91	.95	no	−0.10	0.93	1.00	no
*Abcc8*	0.23	1.17	.93	no	0.20	1.15	1.00	no

Fold change was calculated as the ratio of gene expression in the high glucose state to the low glucose state. Threshold for determining differential expression (DE) was set at ±1 for Log2 Fold Change and 0.05 for adjusted *P* value.

### Low-glucose exposure suppresses mTORC1 activity and promotes β-cell glucose responsiveness

Given the minimal transcriptional response of neonatal islets to low-glucose exposure, we next asked whether nutrient-sensing pathways might contribute to the observed functional changes. mTORC1 has previously been implicated in β-cell maturation and metabolic plasticity (31), so we examined whether low-glucose exposure alters mTORC1 activity in neonatal β-cells.

**Figure 4:**
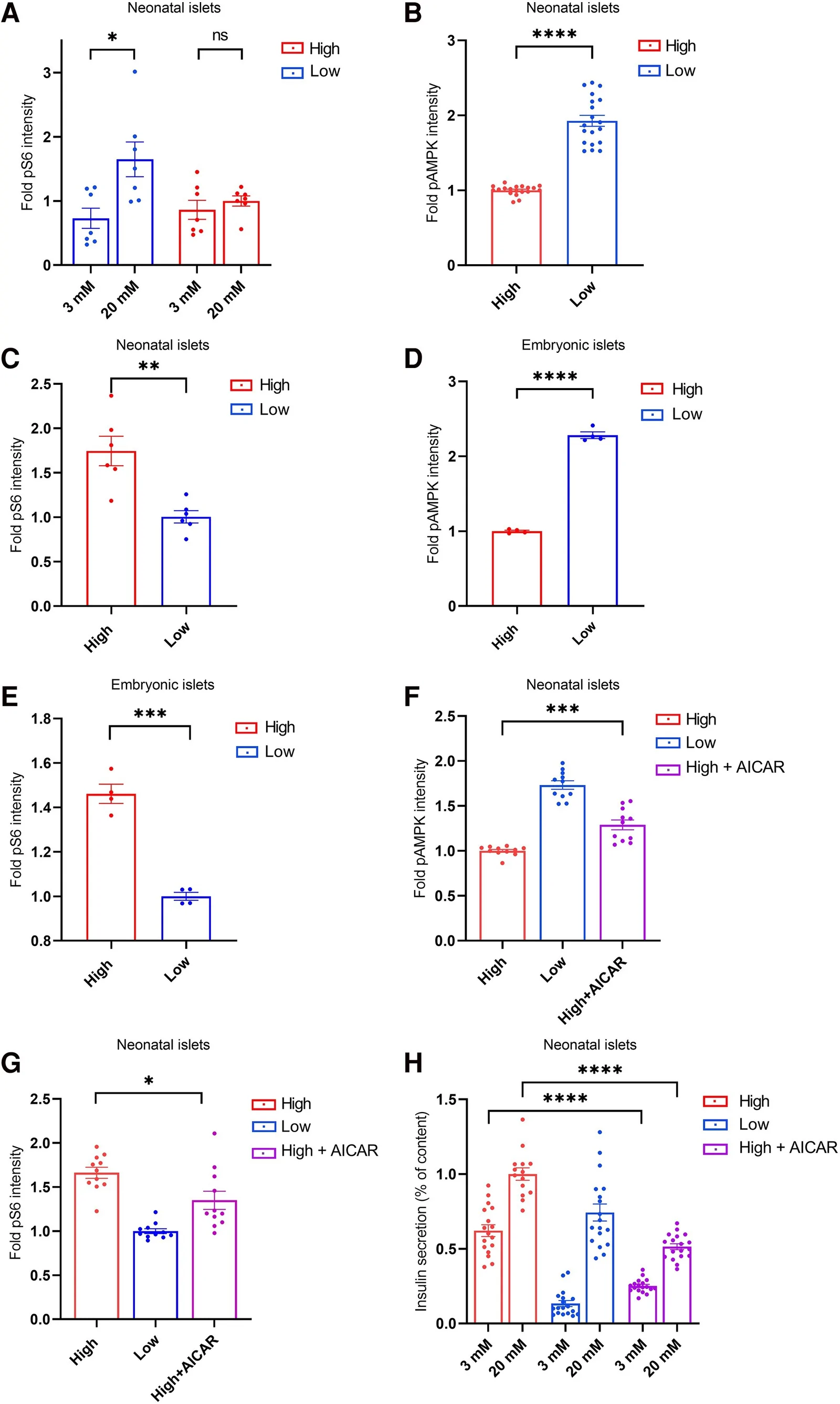
FACS and functional analysis of P1 β-cells under high glucose, low glucose and high glucose + AICAR conditions. **(**A) FACS analysis of pS6 intensity in β-cells from isolated neonatal (P1) mouse islets after 24 hours pretreatment in RPMI medium under high- (11 mM) or low-glucose (3 mM) conditions, followed by incubation with 3 mM or 20 mM glucose in RPMI medium for 1 hour. Each point represents data from a group of 40-50 islets. Results were confirmed in 3 independent experiments (*n* = 6). (B) FACS analysis of pAMPK intensity in β-cells from isolated neonatal (P1) mouse islets after 24 hours pretreatment in RPMI medium under high- (11 mM glucose) or low-glucose (3 mM glucose) conditions. Each point represents insulin measurement from a group of 25-30 islets. Results were confirmed in three independent experiments (*n* = 6). (C) FACS analysis of pS6 (*n* = 6) intensity in beta cells from isolated neonatal (P1) mouse islets after 24 hours pretreatment in RPMI medium under high glucose (11 mM) or low glucose (3 mM) conditions. Each point represents measurements from a group of 25-30 islets in a representative experiment. Results were confirmed in 3 independent experiments. (D) FACS analysis of pAMPK intensity (*n* = 4) in β-cells from isolated embryonic (E18) mouse islets after 24 hours pretreatment in RPMI medium under high glucose (11 mM) or low glucose (3 mM) conditions. Each point represents measurements from a group of 25-30 islets in a representative experiment. Results were confirmed in 3 independent experiments. (E) FACS analysis of pS6 intensity (*n* = 4) in β-cells from isolated embryonic (E18) mouse islets after 24 hours pretreatment in RPMI medium under high glucose (11 mM) or low glucose (3 mM glucose) conditions. Each point represents measurement from a group of 25-30 islets in a representative experiment. Results were confirmed in 3 independent experiments. (F) FACS analysis of pAMPK intensity (*n* = 12) in β-cells from isolated neonatal (P1) mouse islets after 24 hours pretreatment in RPMI medium under high glucose (11 mM), low glucose (3 mM glucose) or high glucose + AICAR (11 mM glucose supplemented with 0.5 mM AICAR) conditions. Each point represents data from a group of 25-30 islets. Results were confirmed in 3 independent experiments. (G) FACS analysis of pS6 intensity (*n* = 9) in β-cells from isolated neonatal (P1) mouse islets after 24 hours pretreatment in RPMI medium under high glucose (11 mM), low glucose (3 mM), or high glucose + AICAR (11 mM glucose supplemented with 0.5 mM AICAR) conditions. Each point represents measurements from a group of 25-30 islets. Results were confirmed in 3 independent experiments. (H) Insulin secretion by neonatal (P1) mouse islets after 24 hours pretreatment in RPMI medium under high glucose (11 mM glucose), low glucose (3 mM glucose) or high glucose + AICAR (11 mM glucose supplemented with 0.5 mM AICAR) conditions, followed by sequential stimulation with 3 and 20 mM glucose in KRBH for 1 hour. Insulin secretion is expressed as a percentage of total insulin content. Each point represents insulin measured from groups of 9 islets (*n* = 6). Results were confirmed in 3 independent experiments. All data are presented as mean ± SEM. Statistical analysis: unpaired Student *t*-test; * *P* < .05, ** *P* < .01, *** *P* < .001, **** *P* < .0001.

To examine the effect of in vitro exposure to low glucose on mTORC1 activity, we used flow cytometry to measure phosphorylation of ribosomal protein S6 (pS6), a downstream target of mTORC1. Whereas P1 islets exposed to high-glucose conditions did not respond to high glucose with increased pS6, low-glucose islets showed significant glucose responsiveness (Fig. 4A). This is consistent with the results obtained for insulin secretion and indicates a possible role of mTORC1 signaling in the acquisition of glucose responsiveness and β-cell maturation.

### Low glucose activates AMPK and suppresses mTORC1 signaling in neonatal β-cells

As AMPK is a key cellular energy sensor and a major upstream regulator of mTORC1 signaling (32), we next examined whether the effects of low-glucose exposure were associated with changes in AMPK activity.

Low-glucose exposure increased AMPK phosphorylation in insulin-positive cells from P1 islets compared with high-glucose conditions (Fig. 4B), indicating AMPK activation. This was accompanied by reduced mTORC1 signaling, as assessed by pS6 (Fig. 4C), and similar changes were observed in E18 islets (Fig. 4D, E). To determine whether AMPK activation is sufficient to mimic the effects of low glucose, high-glucose P1 islets were treated with the AMPK agonist AICAR. AICAR increased pAMPK in high-glucose islets (Fig. 4F), reduced mTORC1 signaling (Fig. 4G), and decreased insulin secretion to levels comparable to those of low-glucose islets (Fig. 4H). These findings indicate that AMPK activation is sufficient to suppress high basal insulin secretion in neonatal β-cells.

### P1 β-cells are depolarized and generate action potentials following exposure to low glucose

**Figure 5:**
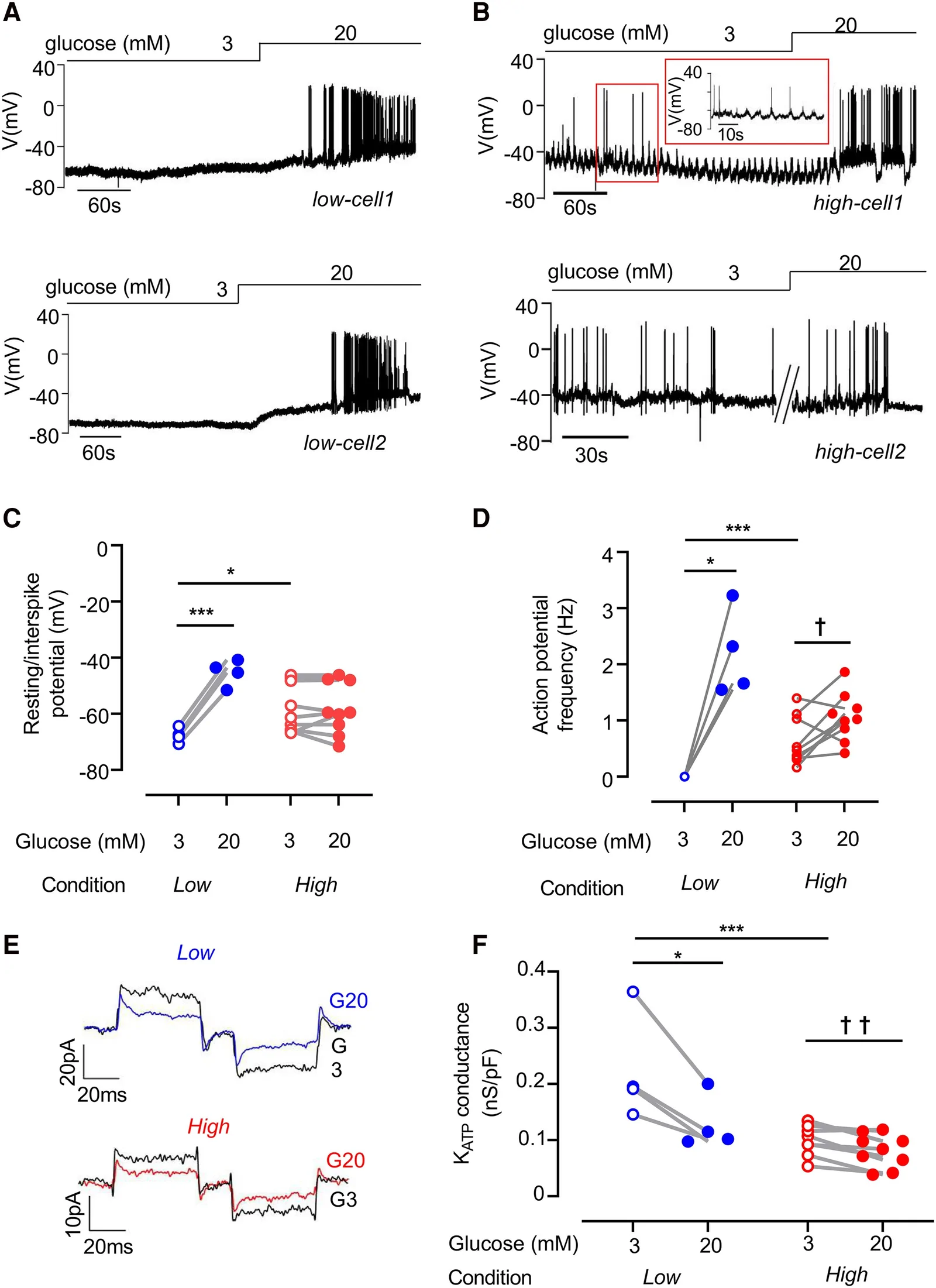
Electrophysiological analysis of P1 high and low glucose β-cells. (A-B) Membrane potential recordings from low- (A) and high-glucose (B) P1 β-cells. Two representative recordings are shown for each condition. Glucose was varied from 3 to 20 mM, as indicated by the staircase above the membrane potential traces. The small baseline transients seen in the top trace of B reflect action potentials in neighboring β-cells that invade the β-cell connected to the recording electrode via the gap junctions. An expanded part of the recording is shown in the inset. Representative measurements from 4 and 9 islet β-cells in islets from 3 mice. (C) Most negative potential at 3 and 20 mM glucose in β-cells of low- and high-glucose P1 islets. Recordings from 4 low-glucose and 9 high-glucose islets. *P < .05 and ***P < .001 for indicated statistical significances (Student *t*-test). (D) As in (C) but showing action potential frequency at 3 and 20 mM glucose. *P < .05 and **P < .01 vs 3 mM glucose in low-glucose β-cells; †*P* < .05 vs 3 mM glucose in high-glucose-cultured β-cells (Student *t*-test). (E) Top: Perforated whole-cell K_ATP_ channel measurements at steady-state in the presence of 3 and 20 mM glucose (G3 & G20) in low- and high-glucose islets as indicated. K_ATP_ channel activity was visualized by application of ±10 mV voltage pulses from a holding potential of −70 mV. (F) Effects of increasing glucose from 3 to 20 mM on K_ATP_ channel conductance normalized to cell size (cell capacitance) in low and high glucose-cultured β-cells. Measurements were made in intact islets from 4 (low) and 9 (high) glucose islets. *P < .05; ††*P* < .01 for indicated statistical significances (Student *t*-test).

Insulin secretion is initiated by action potential firing in β-cells. We performed perforated patch whole-cell measurements in β-cells within intact high or low glucose-incubated P1 islets (Fig. 5A-5D). In high-glucose islets exposed to 3 mM glucose, β-cells had a less negative membrane potential than their low-glucose counterparts (Fig. 5A-5B). This correlated with the occurrence of spontaneous action potentials, whereas low-glucose islet cells were consistently electrically silent. The smaller oscillations between the full-amplitude action potentials represent action potential firing in neighboring β-cells that invade the patch-clamped cell via gap junctions (33). Increasing glucose from 3 to 20 mM led to membrane depolarization in low-glucose β-cells but had little effect in high-glucose cells (Fig. 5C). In low-glucose-cultured β-cells, the mean action potential frequency was dramatically increased when glucose was elevated during the electrophysiological experiments but had little effect in high-glucose-cultured cells (Fig. 5D).

We correlated glucose-induced changes in membrane potential and action potential firing with alterations in K^+^ channel activity. Fig. S2a-b (27) shows the whole-cell current (*I*)–voltage (*V*) relationships recorded during perforated patch whole-cell recordings from β-cells in intact islets exposed to 1 and 10 mM glucose, in response to voltage ramps between −100 and +30 mV. In β-cells exposed to 1 mM glucose, the *I–V* relationship was linear between −100 and −50 mV (34). After increasing glucose to 20 mM, the slope of the *I–V* relationship decreased, and a significant deviation from linearity became evident at membrane potentials >−50 mV, indicating the opening of voltage-gated Ca^2+^ channels (VGCCs). The inset in Fig. S2a (27) shows the responses between −80 and −60 mV on an expanded vertical axis, making it clear that the current crosses the horizontal axis at −78 mV and −58 mV (providing an indirect measure of the β-cell membrane potential at 1 and 10 mM glucose). Fig. S2b (27) summarizes the time-dependent change in whole-cell conductance estimated from the linear parts of the current responses to the voltage ramps (ie, ΔI/ΔV) before and after increasing glucose from 1 to 10 mM, indicating a 50% reduction from ∼0.35 to 0.2 nS/pF (4). We conclude that ±10 mV voltage excursion applied from a holding potential of −70 mV would provide a reliable measure of the whole-cell conductance.

We applied this methodology to β-cells in islets incubated at high and low glucose (Fig. 5E-5F). In low-glucose cells, increasing glucose from 3 to 10 mM reduced resting K^+^ conductance (reflecting K_ATP_ channel activity) by ∼43%, from ∼0.2 to ∼0.1 nS/pF. In high-glucose P1 β-cells, K_ATP_ conductance was low at 3 mM glucose (0.1 nS/pF) and was only marginally inhibited (in absolute terms) by high glucose (Fig. 5E-5F). The low K_ATP_ conductance explains why β-cells in P1 islets cultured in high glucose are spontaneously active at low glucose.

#### Elevated basal [Ca^2+^]_i_ in high glucose P1 islets

**Figure 6:**
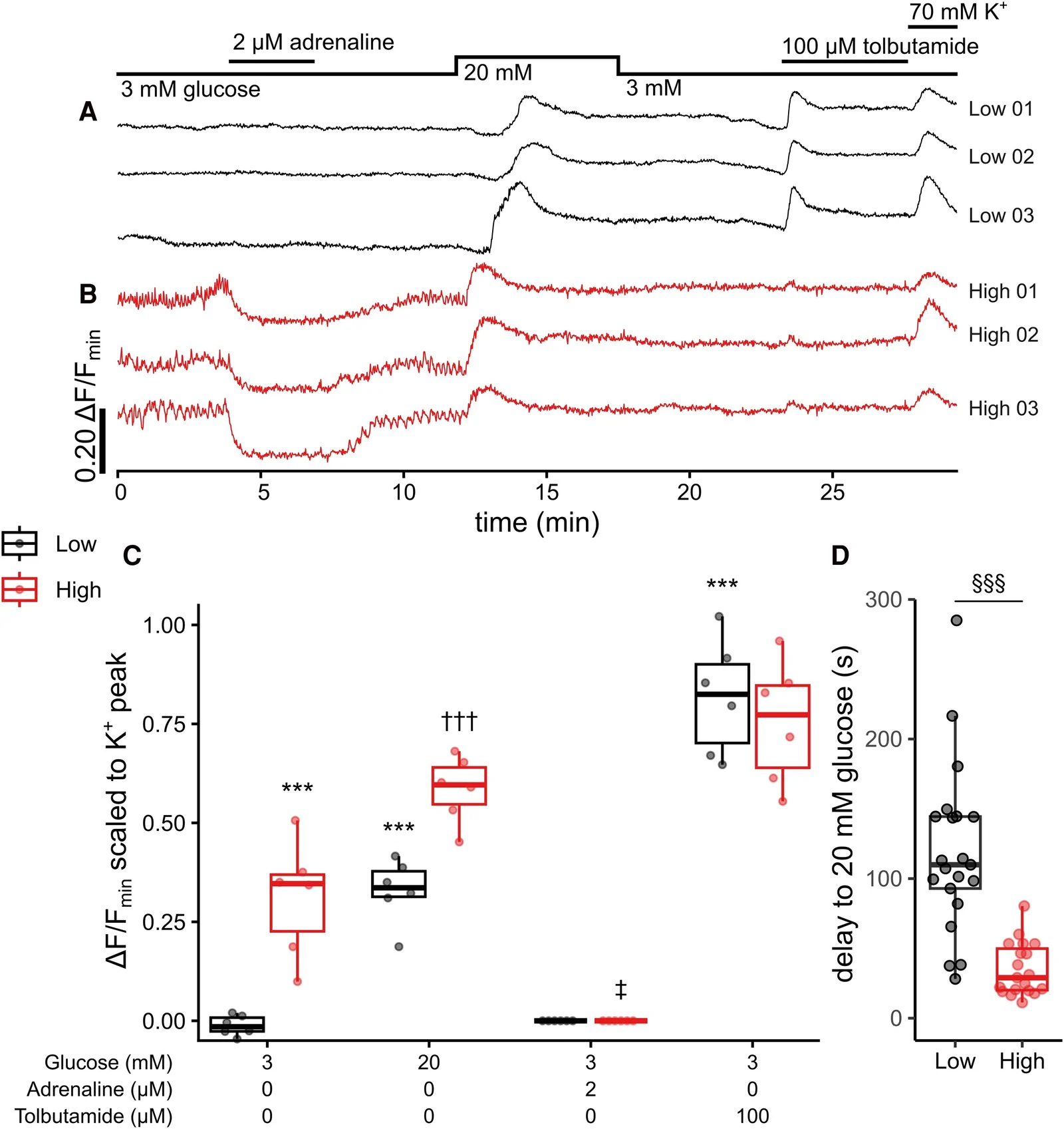
(Ca^2+^)_I_ responses in β-cells of high and low glucose P1 mouse islets. (A-B) β-cell (Ca^2+^)_i_ at 3 mM glucose, 3 mM glucose + 2 µmol/L Adrenaline, 20 mM glucose, 3 mM glucose + 0.2 mM tolbutamide, and 70 mM (K^+^)_o_ as indicated in low (A) and high glucose (B) P1 islets. Three representative traces were selected for display. Experiments were repeated in islets from 4 litters of mice. (C) Bar graphs comparing (Ca^2+^)_i_ in low-glucose islets at low and high-glucose (steady-state) and in the presence of adrenaline or tolbutamide (peak) as indicated. (D) Latency between increasing glucose from 3 to 20 mM and a detectable increase in (Ca^2+^)_i_ in high- and low-glucose islets. Statistical significance indicated by symbols where multiplicity denotes *P*-value thresholds (*, †, ‡, §: *P* < .05; **, ††, ‡‡, §§: *P* < .01; ***, †††, ‡‡‡, §§§: *P* < .001). Comparisons: * vs 3 mM glucose low glucose; † vs 3 mM glucose high glucose (mixed-effects model: Tukey HSD); ‡ vs 3 mM glucose high glucose (*t*-test, raw intensity values); § comparison indicated by line (Mann–Whitney).

Action potential firing culminates in the opening of plasmalemmal VGCCs and so leads to an increase in the cytoplasmic Ca^2+^ concentration ([Ca^2+^]_i_). We explored this by imaging β-cell [Ca^2+^]_i_ dynamics (measured using fluo-4 preloaded into the cells/islets) in intact islets from high- or low-glucose P1 islets (Fig. 6). In low-glucose-incubated β-cells, [Ca^2+^]_i_ at 3 mM glucose was low. Increasing glucose to 20 mM induced a reversible triphasic response consisting of an initial reduction followed by a large increase that decayed to a stable plateau (Fig. 6A). At low glucose, addition of adrenaline had no effect on [Ca^2+^]_i_. The addition of the K_ATP_ channel blocker tolbutamide (0.2 mM) to 3 mM glucose evoked a response similar to that observed at high glucose. Membrane depolarization induced by increasing extracellular K^+^ ([K^+^]_o_) to 70 mM evoked a transient increase in [Ca^2+^]_i_ reflecting the opening and subsequent inactivation of the VGCCs.

Very different results were obtained in β-cells within P1 islets cultured under high-glucose conditions (Fig. 6B). These cells are spontaneously active at 3 mM glucose, seen as a sustained elevation of [Ca^2+^]_i_ and/or rapid [Ca^2+^]_i_ oscillations. Application of adrenaline (2 µM), which acts by activation of α_2_ adrenoceptors (35), led to a prompt and reversible reduction of [Ca^2+^]_i_. Increasing glucose from 3 to 20 mM induced variable increases in [Ca^2+^]_i_, ranging from no effect to a prominent but short-lived increase beyond the high basal level. Addition of the K_ATP_ channel blocker tolbutamide (0.2 mM) in the presence of 3 mM glucose had little effect on [Ca^2+^]_i_, whereas membrane depolarization induced by increasing [K^+^]_o_ to 70 mM transiently increased [Ca^2+^]_i_. In both low and high-glucose-cultured islets, no oscillations in [Ca^2+^]_i_ were observed at 20 mM glucose, probably because of continuous action potential firing (15).

Figure 6C compares steady-state [Ca^2+^]_i_ levels in β-cells from high- and low-glucose P1 islets at 3 mM glucose. Responses have been normalized to the increase in [Ca^2+^]_i_ produced by application of high [K^+^]_o_. The latency between elevating glucose from 3 to 20 mM and the peak [Ca^2+^]_i_ response was 3 times shorter in high-glucose β-cells than in low-glucose β-cells (Fig. 6D).

### Diazoxide lowers insulin secretion in high-glucose P1 islets

**Figure 7:**
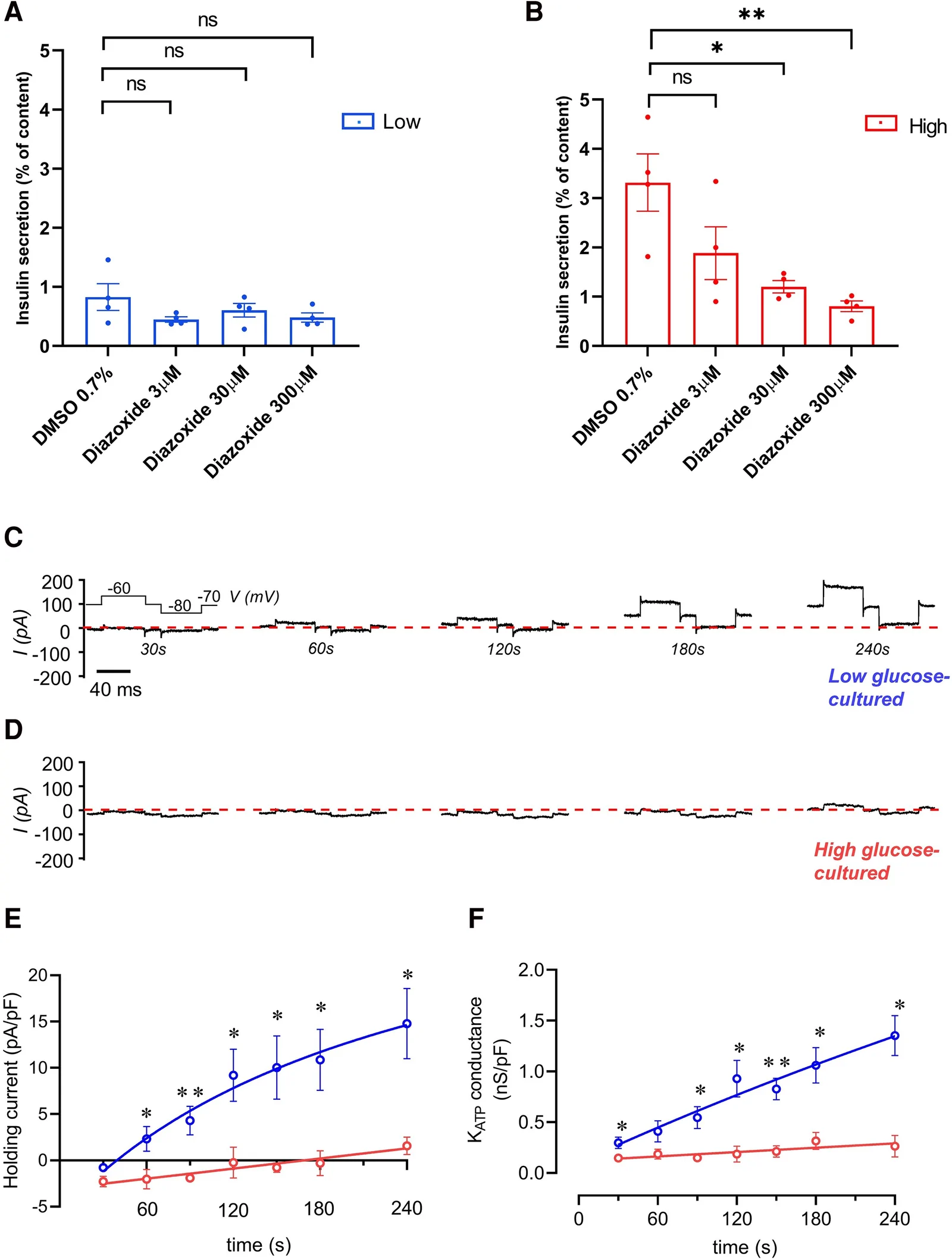
Inhibition of basal insulin secretion in low glucose P1 islets reflects increased K_ATP_ channel density. (A) Insulin secretion by neonatal (P1) mouse islets preincubated in low glucose followed by 1 hour incubation in KRBH with different concentrations of diazoxide. Insulin secretion is expressed as a percentage of total insulin content. Each point represents insulin measured in groups of 9 islets (*n* = 4). (B) Insulin secretion by neonatal (P1) mouse islets preincubated in high glucose followed by 1 hour incubation in KRBH with different concentrations of diazoxide. Insulin secretion is expressed as a percentage of total insulin content. Each point represents insulin measurements in groups of 9 islets (*n* = 4). All data are presented as mean ± SEM. Statistical analysis: unpaired Student *t*-test; * *P* < .05, ** *P* < .01, *** *P* < .001, **** *P* < .0001. (C-D) Standard whole-cell K_ATP_ conductance measured in low (C) and high glucose-cultured (D) β-cells in intact P1 mouse islets monitored by the current responses (I) to application of ±10 mV voltage (*V*) pulses from a holding potential of −70 mV (indicated by staircase above current trace). Plasmalemmal K_ATP_ channels were activated by dialyzing the cell interior with 0.1 mM ATP and 0.3 mM ADP (Mg-salts). Representative measurements from 8 and 10 β-cells in islets from 4 mice. The dashed lines indicate the zero current level. (E-F) Whole-cell holding current (E) and G_K,ATP_ conductance (F) measured at different times after establishing the whole-cell configuration. *P < .05; **P < .01 vs high glucose incubated islets. Data are mean values ± SEM for seven cells in each group.

We reasoned that the occurrence of spontaneous action potential firing and [Ca^2+^]_i_ oscillations in high-glucose islets incubated at 3 mM glucose results from decreased K_ATP_ channel activity. Consistent with this, treatment of high-glucose islets with the K_ATP_ channel activator diazoxide produced a dose-dependent inhibition of insulin secretion (Fig. 7B), whereas basal insulin secretion was low in low-glucose islets and not affected by diazoxide (Fig. 7A).

### Reduced plasmalemmal K_ATP_ channel density in high glucose P1 β-cells

The whole-cell K_ATP_ conductance (G_K,ATP_) is the product γ_KATP_*N*P_open_, where γ_K,ATP_ is the single-channel conductance, *N* is the number of channels in the plasma membrane, and *P*_open_ is the open probability. To determine which of these parameters is affected by low- and high-glucose culture, we performed standard whole-cell measurements in which the chemical composition of the cytoplasm was “clamped” by the electrode-filling solution. We measured the increase in G_K,ATP_ when the cell interior was dialyzed through the recording electrode with an intracellular medium in which ATP and ADP were held at 0.1 and 0.3 mM, respectively. These conditions are associated with strong (maximal) activation of the K_ATP_ channels whilst minimizing the rundown of channel activity observed with ATP-free intracellular media (15). Figure 7C-7D shows the increases in G_K,ATP_ measured during the first 4 minutes after establishment of the whole-cell configuration in β-cells of low- and high-glucose-incubated P1 islets. Figure 7E summarizes these responses. Figure 7F shows the time-dependent changes in the holding current measured at −70 mV. In the high glucose-incubated β-cells it is below zero for the initial 120 s, suggesting the membrane potential in these cells is less negative than −70 mV. For both G_K,ATP_ and the holding current, the changes in low-glucose-incubated β-cells are faster and larger than in high-glucose-incubated β-cells. G_K,ATP_ measured 4 minutes (240 s) after establishment of the whole-cell configuration was 80% lower in β-cells from high- than low-glucose-pretreated islets. This suggests that the plasmalemmal K_ATP_ channel density is reduced in high-glucose-incubated P1 β-cells.

## Discussion

Insulin serves a dual role as both a growth factor and a key regulator of metabolic homeostasis. During embryonic and early postnatal development, insulin supports tissue growth and anabolic processes (36, 37). As development proceeds, its primary physiological function shifts toward maintaining glucose homeostasis by regulating glucose uptake and storage in insulin-sensitive tissues (38).

During the early postnatal period, circulating glucose levels are significantly lower than in adults (39), reflecting the distinct metabolic environment of neonatal life. These conditions pose a physiological challenge: β-cells must provide sufficient insulin to support growth despite relatively low circulating glucose levels, while avoiding excessive insulin release that could exacerbate hypoglycemia. These constraints suggest that neonatal β-cells possess mechanisms that maintain insulin secretion even under limited glucose supply. Here, we describe a mechanism that accounts for this adaptive regulation of insulin secretion in the neonatal metabolic environment.

The effects of prior high-glucose incubation reversed with a time constant of 1.5 hours. This is much slower than the reversal of glucose-induced changes in electrical activity, [Ca^2+^]_i_, and insulin secretion observed in adult islets, which typically occurs within minutes. However, this is likely too fast to involve changes in gene transcription. This is consistent with our RNA-seq data from neonatal islets, which show that only a small number of genes are differentially expressed (Fig. 3B), instead highlighting post-translational or membrane-trafficking events.

Neonatal β-cells exhibit heightened mTORC1 activity that remains elevated even under low-glucose conditions, consistent with the high anabolic demands of early postnatal growth (31). Sustained mTORC1 signaling promotes growth-associated metabolic programs and delays the transition to mature glucose responsiveness, a pattern also observed in diabetic β-cells (40). Inhibiting S6 kinase, a downstream effector of mTORC1, has been shown to partially reverse the detrimental effects of chronic hyperglycemia on β-cell function (41).

In our study, mTORC1 activity in high-glucose neonatal islets remains unchanged between low (3 mM) and high (20 mM) glucose, indicating a lack of glucose-dependent regulation. In contrast, low-glucose conditions induce a significant increase in pS6 levels in response to high glucose (Fig. 4A), suggesting that nutrient withdrawal sensitizes islets to glucose and promotes mTORC1 responsiveness. The emergence of glucose-dependent mTORC1 regulation after low-glucose exposure suggests a shift toward a more metabolically responsive state rather than full maturation. Consistent with this, mTORC1 integrates nutrient signals with transcriptional programs that control β-cell growth, metabolism, and functional identity (42). Together, these findings suggest that although mTORC1 contributes to β-cell maturation, persistent unmodulated mTORC1 activity—observed in both neonatal and diabetic β-cells—may reflect a distinct metabolic state rather than developmental immaturity. This state supports high anabolic demand and enables neonatal β-cells to rapidly adjust insulin secretion to changing nutrient availability, balancing postnatal growth requirements with protection against hypoglycemia. mTORC1 has been linked to the maintenance of glycolytic programs that favor aerobic glycolysis over oxidative metabolism, consistent with reduced metabolic flexibility. This configuration may correspond to a Warburg-like metabolic state that limits oxidative capacity and functional responsiveness (43). In pancreatic β-cells, AMPK activation has been linked to the regulation of K_ATP_ channel trafficking, promoting the recruitment of these channels to the plasma membrane (44). An increased density of functional K_ATP_ channels at the cell surface stabilizes the resting membrane potential, reduces membrane excitability, and thereby limits insulin secretion under low-glucose conditions (45). Through this mechanism, AMPK serves as a molecular link between cellular energy status and β-cell electrical activity.

In our experiments, we examined P1 neonatal islets under two metabolic conditions: the high-glucose group (24 hours in 11 mM glucose medium) and the low-glucose group (3 mM glucose for the same duration). Our data indicate that in β-cells from neonatal high-glucose islets, AMPK activity is low (Fig. 4B), and the activity and surface density of K_ATP_ channels are reduced (Fig. 5F, H), leading to membrane depolarization (Fig. 5B), action potential firing, and elevation of [Ca^2+^]_i_ under hypoglycemic conditions (Fig. 6A). This explains the elevated basal (at 3 mM glucose) insulin secretion in high-glucose P1 islets (Fig. 1C). Because β-cells in the high-glucose group are already closer to the depolarization threshold, relatively small increases in glucose concentration may more readily trigger calcium influx and insulin secretion, explaining the left-shift of the glucose-response profile observed in Fig. 1E.

In contrast, β-cells from low-glucose islets exhibit high AMPK activity (Fig. 4B) and have higher activity and surface density of K_ATP_ channels at low glucose (Fig. 5F, and 7D). As a result, the β-cells are hyperpolarized and electrically silent (Fig. 5A). Consequently, intracellular [Ca^2+^]i is low (Fig. 6B), explaining the low rate of insulin secretion (Fig. 1C), echoing the functional state observed in adult islets.

Comparing whole-cell values of G_K,ATP_ for standard and perforated patches, we estimate that K_ATP_ channel activity is inhibited by 80% in intact P1 β-cells at 3 mM glucose, regardless of the glucose concentration during prior exposure. A similar decrease is derived when β-cells were dialyzed with a low ATP-high ADP mixture to maximally activate the K_ATP_ channels. The lower G_K,ATP_ value (resulting from an 80% reduction of the plasmalemmal K_ATP_ channel density) also explains the lower threshold for insulin secretion in high-glucose islets. Because K_ATP_ channel activity is already reduced, a smaller increase in the cytoplasmic ATP/ADP ratio is sufficient to evoke action potential firing and insulin secretion.

Together, our data indicate that changes in glucose availability during the early postnatal period acutely modulate the functional state of neonatal β-cells and shape their pattern of insulin secretion. This effect is mediated, at least in part, by AMPK/mTOR signaling, which regulates K_ATP_ channel activity or surface density. The finding that pharmacological activation of AMPK in high-glucose neonatal islets using AICAR (Fig. 4F) decreased basal insulin secretion (Fig. 4H) supports this concept.

## Conclusion

Our data demonstrate that changes in glucose availability are sufficient to acutely and reversibly modulate insulin secretion and glucose responsiveness in neonatal β-cells. We show that ∼1 hour low glucose exposure suppresses basal insulin secretion, raises the glucose threshold, and establishes glucose-dependent regulation of mTOR and AMPK signaling. These changes occur rapidly and without large-scale transcriptional remodeling, indicating that metabolic cues alone can directly regulate key aspects of β-cell function at the level of stimulus-secretion coupling. In this context, acute metabolic regulation may help balance the anabolic demands of early growth with the need to avoid life-threatening hypoglycemia. More broadly, these findings highlight the importance of nutrient-sensing pathways in defining β-cell functional states and suggest that the hypoglycemic environment of the early postnatal period may contribute to the acquisition of β-cell glucose responsiveness. These findings suggest that neonatal and adult β-cells operate at distinct metabolic set points tuned to their physiological demands. Within this framework, the transient hypoglycemic environment after birth may act as a contextual signal that promotes the progressive establishment of adult-like glucose responsiveness. Given that impaired nutrient sensing and glucose responsiveness are hallmarks of β-cell dysfunction in type 2 diabetes, our findings raise the possibility that targeted modulation of nutrient-sensing pathways—particularly the AMPK/mTOR axis—could help restore glucose sensitivity in dysfunctional β-cells.

## Data Availability

The RNA-sequencing data generated in this study have been deposited in the NCBI Gene Expression Omnibus (GEO) under accession number GSE314732 listed in “References” (30). The supplementary material associated with this article has been deposited in the Weizmann Institute Repository and is available at (27). All other data supporting the findings of this study are available from the corresponding authors upon reasonable request.
